# Three new species of *Sesioctonus* Viereck (Hymenoptera, Braconidae, Agathidinae) from Peru

**DOI:** 10.3897/zookeys.196.3086

**Published:** 2012-05-21

**Authors:** Lidia Sulca, Michael J. Sharkey

**Affiliations:** 1 Natural History Museum, University of San Marcos, Lima, Peru; 2 Department of Entomology, University of Kentucky, Lexington, Kentucky, 40502, USA

**Keywords:** Insecta, taxonomy, biodiversity, parasitoid

## Abstract

Three new species of *Sesioctonus* (Braconidae: Agathidinae) are described and illustrated, i.e., *Sesioctonus huggerti*, *Sesioctonus wayquecha*, and *Sesioctonus bina*. Two new Peruvian species records for *Sesioctonus* are reported: *Sesioctonus longinoi* and *Sesioctonus diazi*. A revised key to all known species of *Sesioctonus* is presented.

## Introduction

*Sesioctonus* Viereck, 1912 is a Neotropical genus of Agathidinae which for which the biology is largely unknown, only *Sesioctonus parathyridis* is recorded as a larval parasitoid of *Parathyris perspicilla* Stall (Lepidoptera: Arctiidae) (Viereck 1912). Briceño (2003) revised the species of *Sesioctonus* and included twenty six species and Sharkey and Briceño (2005) described five new species. Here we describe three new species from Peru and two additional species are reported for the first time from Peru.

## Methods

Morphological terminology follows that of Sharkey and Wharton (1997). Figures in this paper that are followed by the letter ‘B’ refer to those in Briceño (2003). The species descriptions are of the holotypes with variation given in parenthesis.

Unless otherwise stated specimens are deposited in the Natural History Museum, University of San Marcos, Lima, Peru (MUSM), with duplicates deposited in the Hymenoptera Institute Collection at the University of Kentucky, USA (HIC).

## Results and discussion

### Diagnosis

Members of *Sesioctonus* are restricted to the Neotropical realm of the New World and may be distinguished from all other agathidine braconids with the following combination of characters: Mesoscutum smooth, lacking notauli; tarsal claws simple, lacking a basal claw; hind coxal cavities open, sharing a common opening with the metasomal foramen.

### Key to *Sesioctonus* species of the world, modified from Sharkey and Briceño (2003)

**Table:** 

1	Occipital tubercles present (Figs 16B–18B)	2
–	Occipital tubercles absent. (Figs 19B)	17
2(1)	Epicnemial carina straight medially or absent, not indented at midline, between fore coxae) (Figs 4B, 23B)	3
–	Epicnemial carina bilobed medially (indented at midline, between fore coxae) (Figs 3B, 22B)	6
3(2)	Epicnemial carina complete laterally (Figs 3B, 22B)	4
–	Epicnemial carina incomplete or absent laterally (Fig. 23B)	5
4	(3) Interantennal space with longitudinal rounded keel; face without median longitudinal carinae	*Sesioctonus garciai* Briceño
–	Interantennal space lacking longitudinal keel; face with median longitudinal carinae	*Sesioctonus huggertii* sp. n.
5(3)	Face with median longitudinal carina (Fig. 13B)	*Sesioctonus acrolophus* Briceño
–	Face without median longitudinal carina (similar to Figs 12B, 14B)	*Sesioctonus analogus* Briceño
6(3)	Mid coxa color variable, but not completely melanic	7
–	Mid coxa completely melanic	10
7(6)	Fore wing banded from base: yellow, black, yellow, black	*Sesioctonus chaconi* Briceño
–	Fore wing infuscate (melanic)	8
8(7)	Fore tibia with spines; mid femur yellowish orange	9
–	Fore tibia without spines; mid femur melanic	*Sesioctonus longinoi* (part) Sharkey & Briceño
9(8)	Median longitudinal carinae of propodeum absent, median areola of metanotum with lateral carinae not meeting posteriorly, subpronope triangular	*Sesioctonus peruviensis* Briceño
–	Median longitudinal carinae of propodeum present, median areola of metanotum with lateral carinae meeting posteriorly, subpronope oval	*Sesioctonus bina* sp. n.
10(6)	Longitudinal carina(e) of scutellar depression present and fore wing banded from base: yellow, black, yellow, black	*Sesioctonus venezuelensis* Briceño
–	Longitudinal carina(e) of scutellar depression absent and/or fore wing not banded	11
11(10)	Mesoscutum black; median areola of metanotum with longitudinal rugosities (Fig.29B); median tergite of first metasomal segment without pair of lateral longitudinal carina (similar to Fig. 34B); fore wing (RS+M)a vein complete (Fig. 10aB)	*Sesioctonus kompsos* Briceño
–	Mesoscutum yellowish orange, or if black then not combining other characters	12
12(11)	Mesoscutum melanic	13
–	Mesoscutum yellowish orange	14
13(12)	Fore wing infuscate with large hyaline spot; metasoma reddish brown except last few segments melanic	*Sesioctonus brasiliensis* Briceño
–	Fore wing either infuscate without hyaline spot or hyaline basally and infuscate apically; metasoma yellowish orange* S. dichromus* Briceño
14(12)	Median longitudinal carina of propodeum present and complete	*Sesioctonus ariasi* Briceño
–	Median longitudinal carina of propodeum absent or incomplete	15
15(14)	Subpronope triangular, three sides almost equal (Fig. 1B); fore wing 3RSa vein absent (Fig. 10B)	*Sesioctonus boliviensis* Briceño
–	Subpronope more oval-shaped, weakly triangular with vertical sides longer than dorsal side (Fig. 2B); fore wing 3RSa vein present (Fig. 9B)	16
16(15)	Fore wing banded from base: yellow, black, yellow, black* S. diazi* Briceño
–	Fore wing infuscate (melanic)	*Sesioctonus longinoi* (part) Sharkey & Briceño
17(1)	Occiput excavated (similar to Figs 16B–18B)	*Sesioctonus eumenetes* Briceño
–	Occiput not excavated (Fig. 19B)	18
18(17)	Interantennal space without sharp longitudinal keel	19
–	Interantennal space with sharp longitudinal keel (Fig. 11B)	34
19(18)	Basal sterna of metasoma chalk-white	20
–	Basal sterna of metasoma not chalk-white, rather melanic or yellowish orange	22
20(19)	Head orange (Fig. 1dB	*Sesioctonus susanai* Sharkey & Briceño
–	Head black	20
21(20)	Fore and hind coxa pale yellow (Fig. 1bB)	*Sesioctonus stephaniai* Sharkey & Briceño
–	Fore and hind coxa melanic (Fig. 1aB)	*Sesioctonus philipi* Sharkey & Briceño
22(19)	Median areola of metanotum with lateral carinae meeting posteriorly (Figs 25B, 26B)	23
–	Median areola of metanotum with lateral carinae absent or, if present, not meeting posteriorly (Figs 27B, 28B)	32
23(22)	Epicnemial carina present (Figs 3B, 4B)	24
–	Epicnemial carina absent	28
24(23)	Epicnemial carina complete laterally (Fig. 3B)	25
–	Epicnemial carina incomplete laterally (Fig. 4B)	30
25(24)	Hind tibia entirely melanic* S. amazonensis* Briceño
–	Hind tibia mostly yellowish orange	26
26(25)	Propodeum with central areola absent	27
–	Propodeum with central areola present	*Sesioctonus areolatus* Briceño
27(26)	Antenna with more than 29 flagellomeres; interantennal space with rounded longitudinal keel (similar to Fig. 12B); hind tibia yellowish orange in basal half, melanic apically	*Sesioctonus miyayensis* Briceño
–	Antenna with less than 28 flagellomeres; interantennal space without longitudinal keel; hind tibia mostly yellowish orange, melanic apically* S. clavijoi* Briceño
28(23)	Scutellar depression with longitudinal carinae; body color yellow, white, and black (Fig. 1cB)* S. torresi* Sharkey & Briceño
–	Scutellar depression without longitudinal carinae; body color yellowish orange and black	29
29(28)	(RS+M)a vein of fore wing complete, median tergite of first metasomal segment with pair of lateral longitudinal carinae	*Sesioctonus ammosakron* Briceño
–	(RS+M)a vein fore wing incomplete, median tergite of first metasomal segment without pair of lateral longitudinal carinae	*Sesioctonus wayquecha* sp. n.
30 (24)	Epicnemial carina straight medially (between fore coxae) (Fig. 4B); body length less than 3mm* S. dominicus* Briceño
–	Epicnemial carina bilobed medially (indented at midline, between fore coxae) (Fig. 3B); body length more than 3mm	31
31(30)	Fore wing (RS+M)a vein complete (Fig. 10aB)* S..armandoi* Briceño
–	Fore wing (RS+M)a vein incomplete (Fig. 9aB)	*Sesioctonus biospleres* Briceño
32(22)	Epicnemial carina present, complete or incomplete laterally (Figs 3B, 4B)	33
–	Epicnemial carina completely absent	*Sesioctonus chrestos* Briceño
33(32)	Fore wing banded, yellow, black, yellow, black; labial palpus 3-segmented	*Sesioctonus galeos* Briceño
–	Fore wing infuscate; labial palpus 4-segmented	*Sesioctonus theskelos* Briceño
34(18)	Third and fourth labial palpomeres not fused; first metasomal median tergite with depression posteriad spiracle (Figs 36B, 37B)* S. grandis* Briceño
–	Third and fourth labial palpomeres fused, first metasomal median tergite with or without depression posteriad spiracle	35
35(36)	First metasomal median tergite with depression posteriad spiracle (similar to Figs 3, 36)	*Sesioctonus qui* Briceño
–	First metasomal median tergite without depression posteriad spiracle	*Sesioctonus parathyridis* Viereck

### New Species Descriptions

#### Sesioctonus huggerti

Sulca & Sharkey sp. n.

urn:lsid:zoobank.org:act:A198E0BC-7DFE-42CB-B5AD-9DF7E63597E6

http://species-id.net/wiki/Sesioctonus_huggerti

Figure 1

##### Diagnosis.

Distinguished from all other known species of *Sesioctonus* by the following suite of characters: Interantennal space lacking longitudinal keel, epicnemial carinae straight medially.

##### Description.

♀ *Length*. Length of body, excluding ovipositor, 5 mm.

*Head*. Flagellum with 30 flagellomeres. Interantennal space lacking longitudinal keel. Antennal sockets moderately excavated. Face with median longitudinal carina. Gena not expanded posteroventrally. Occipital tubercles present. Occiput not excavated. Mandible concave. Outer tooth of mandible not longer than inner tooth. Maxillary palpus with 4 palpomeres. Third and fourth labial palpomeres not fused. *Mesosoma*. Subpronope elongate-oval. Longitudinal carinae of scutellar depression absent. Scutellum convex. Median areola of metanotum smooth, with median longitudinal carina, and with lateral carinae present and not meeting posteriorly. Propodeum convex. Median longitudinal carina of propodeum absent. Epicnemial carina complete, sharp, straight medially (between fore coxae). Hind femur 6 times as long as wide. (RS+M)a vein of fore wing incomplete. 3RSa vein of fore wing absent. 2–1A vein of hind wing not tubular. Cub vein of hind wing not tubular. *Metasoma*. Median tergite of first metasomal segment without pair of lateral longitudinal carinae. Hind wing with 4 hamuli. First metasomal median tergite without depression posteriad spiracle. Length/width ratio of first metasomal median tergite 0.63. Ovipositor 4 mm.

*Color*. Head melanic. Maxillary palpomeres melanic. Labial palpomeres melanic. Pronotum melanic. Mesoscutum yellowish orange. Scutellum yellowish orange. Metanotum yellowish orange. Propodeum melanic. Propleuron melanic. Mesopleuron yellowish orange. Metapleuron melanic. Fore coxa melanic. Fore trochanter melanic. Fore trochantellus melanic. Fore femur melanic. Fore tibia melanic. Fore tarsus melanic. Mid coxa melanic. Mid trochanter melanic. Mid trochantellus melanic. Midfemur melanic. Mid tibia melanic. Mid tarsus melanic. Hind coxa melanic. Hind trochanter melanic. Hind trochantellus melanic. Hind femur melanic. Hind tibia melanic. Hind tarsus melanic. Fore wing entirely infuscate. Stigma melanic. Hind wing entirely infuscate. First metasomal tergum melanic. Second metasomal tergum melanic. Third metasomal tergum melanic. Fourth metasomal tergum melanic. Fifth to eighth metasomal terga melanic. Ovipositor yellowish orange.

♂ Unknown.

*Etymology*. Named in honor of the late Lars Huggert who collected the type specimen.

*Holotype*. PERU, Madre de Dios, Puerto Maldonado, 6–11.i.1984, L. Huggert Leg. (Canadian National Collection).

**Figure 1.**
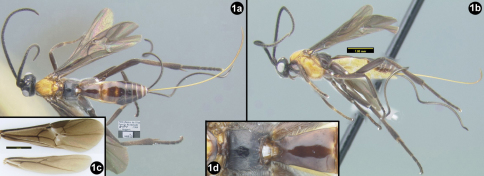
*Sesioctonus huggerti*. **1a** dorsal habitus **1b** lateral habitus **1c** wings **1d** propodeum and first metasomal segment.

##### Distribution.

Known only from the type locality in Peru.

#### Sesioctonus wayquecha

Sulca & Sharkey sp. n.

urn:lsid:zoobank.org:act:19BD24A0-162D-405A-8BF0-5CDA62C5FE86

http://species-id.net/wiki/Sesioctonus_wayquecha

Figure 2 a,b,c,d

##### Diagnosis.

Distinguished from all other known species of *Sesioctonus* by the following suite of characters: occipital tubercles absent, epicnemial carina completely absent, antennal socket not excavated, gena moderately expanded posteroventrally .

##### Description.

♀ *Length*. Length of body, excluding ovipositor, 4.3–5.5 mm.

*Head*. Flagellum with 31 flagellomeres. Interantennal space lacking longitudinal keel. Antennal sockets not excavated. Face without median longitudinal carina. Gena moderately expanded posteroventrally. Occipital tubercles absent. Occiput not excavated. Mandible concave. Outer tooth of mandible not longer than inner tooth. Maxillary palpus with 5 palpomeres. Third and fourth labial palpomeres not fused. *Mesosoma*. Subpronope elongate-oval. Longitudinal carinae of scutellar depression absent. Scutellum convex. Median areola of metanotum smooth, without median longitudinal carina, and with lateral carinae present and meeting posteriorly. Propodeum convex. Median longitudinal carina of propodeum absent. Epicnemial carina completely absent. Fore tibial spines present. Mid tibia with 7 spines. Hind tibia with 15 spines. Hind femur 3.3–4 times as long as wide. (RS+M)a vein of fore wing complete. 3RSa vein of fore wing absent. 2–1A vein of hind wing not tubular. Cub vein of hind wing not tubular. *Metasoma*. Median tergite of first metasomal segment without pair of lateral longitudinal carinae. Hind wing with 4 hamuli. First metasomal median tergite without depression posteriad spiracle. Length/width ratio of first metasomal median tergite 0.63. Ovipositor 0.5–0.6 mm.

*Color*. Head melanic. Antenna melanic. Maxillary palpomeres yellowish orange. Labial palpomeres yellowish orange. Pronotum melanic. Mesoscutum melanic. Scutellum melanic. Metanotum melanic. Propodeum mostly yellowish orange with melanic spots. Propleuron mostly melanic with yellowish orange areas. Mesopleuron melanic. Metapleuron yellowish orange. Fore coxa yellowish orange. Fore trochanter yellowish orange. Fore trochantellus yellowish orange. Fore femur yellowish orange. Fore tibia melanic with yellowish orange ends. Fore tarsus melanic. Mid coxa yellowish orange. Mid trochanter yellowish orange. Mid trochantellus yellowish orange. Mid femur yellowish orange. Mid tibia melanic. Mid tarsus melanic. Hind coxa yellowish orange. Hind trochanter yellowish orange. Hind trochantellus yellowish orange. Hind femur yellowish orange. Hind tibia melanic with a yellow orange apical spot. Hind tarsus melanic. Fore wing entirely infuscate. Stigma melanic. Hind wing entirely infuscate. First metasomal tergum yellowish orange. Second metasomal tergum yellowish orange. Third metasomal tergum yellowish orange. Fourth metasomal tergum yellowish orange but median tergum melanic. Fifth to eighth metasomal terga melanic. Ovipositor yellowish orange.

♂. As in the female (above).

*Etymology*. Named after the type locality, Wayquecha which means ‘brother’ in Quechua.

*Holotype*. PERU. ♀,Cusco, Wayquecha, 13°11'21"S, 71°35'04"W 2837m ,6– 20.x.2007, C. Castillo. Leg.

*Paratypes*: PERU: Cusco: 2♀♀,Wayquecha, 13°11'21"S, 71°35'4"W, 2837m, Malaise, 20.x.2007, C. Castillo Leg.; 3♀♀, 1♂,Wayquecha, 13°10'31"S, 71°34'53"W, 2692m, Malaise, 10.ix.2007, C. Castillo Leg.; ♀, Wayquecha 13°11'S, 71°35'W, 2800m, sweep, 12.ix.2007, C. Castillo Leg; ♂ Wayquecha, 13°10'31"S, 71°34'53"W, 2692m, Malaise, 22.x.2007, C. Castillo Leg.

**Figure 2.**
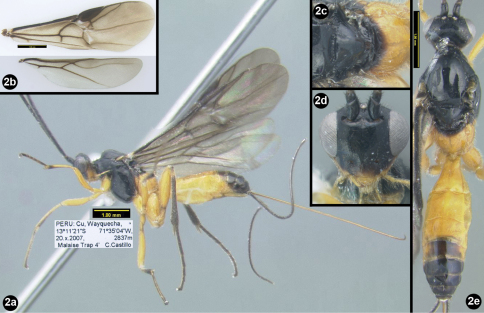
*Sesioctonus wayquecha*. **2a** lateral habitus **2b** wings **2c** dorsal scutellum and propodeum **2d** anterior head **2e** dorsal habitus.

##### Distribution.

Known only from one locality in Peru.

#### Sesioctonus bina

Sulca & Sharkey sp. n.

urn:lsid:zoobank.org:act:5AE5EEED-ACF8-47D4-8189-531FFDBB1209

http://species-id.net/wiki/Sesioctonus_bina

Figure 3

##### Diagnosis.

Distinguished from all other known species of *Sesioctonus* by the following suite of characters: occiput not excavated, subpronope oval, median tergite of first metasomal segment with pair of lateral longitudinal carinae.

##### Description.

♀ *Length*. Length of body, excluding ovipositor, 3.35 mm. Flagellum broken after flagellomere 28. Interantennal space with longitudinal rounded keel. Antennal sockets moderately excavated. Face without median longitudinal carina. Gena not expanded posteroventrally. Occipital tubercles present. Occiput not excavated. Mandible concave. Outer tooth of mandible not longer than inner tooth. Maxillary palpus with 4 palpomeres. *Mesosoma*. Subpronope oval. Longitudinal carinae of scutellar depression absent. Scutellum convex. Median areola of metanotum smooth, without median longitudinal carina, and with lateral carinae present and meeting posteriorly. Propodeum convex. Median longitudinal carina of propodeum present. Epicnemial carina complete, blunt, bilobed medially (between fore coxae). Fore tibial spines present. Mid tibia with 3 spines. Hind tibia with 12 spines. Hind femur 4 times as long as wide. (RS+M)a vein of fore wing incomplete. 3RSa vein of fore wing present. 2–1A vein of hind wing tubular. Cub vein of hind wing not tubular. *Metasoma*. Median tergite of first metasomal segment with pair of lateral longitudinal carinae. Hind wing with 3 hamuli. First metasomal median tergite without depression posteriad spiracle. Length width ratio of first metasomal median tergite 0.5. Ovipositor 1.68 mm.

*Color*. Head black and yellowish orange. Antenna melanic. Maxillary palpomeres yellowish orange. Labial palpomeres yellowish orange. Pronotum melanic. Mesoscutum melanic. Scutellum melanic. Metanotum melanic. Propodeum melanic. Propleuron melanic. Mesopleuron melanic. Metapleuron melanic. Fore coxa yellowish orange. Fore trochanter yellowish orange. Fore trochantellus yellowish orange. Fore femur yellowish orange. Fore tibia yellowish orange. Fore tarsus mostly yellowish orange, but apical tarsomere melanic. Mid coxa yellowish orange. Mid trochanter yellowish orange. Mid trochantellus yellowish orange. Mid femur yellowish orange. Mid tibia yellowish orange in basal half, melanic apically, or yellowish orange basally, otherwise melanic. Mid tarsus melanic. Hind coxa melanic. Hind trochanter melanic. Hind trochantellus melanic. Hind femur melanic. Hind tibia melanic in basal and apical third, yellowish orange medially. Hind tarsus melanic. Fore wing entirely infuscate. Stigma melanic. Hind wing entirely infuscate. First metasomal tergum melanic. Second metasomal tergum yellowish orange but median tergite melanic. Third metasomal tergum melanic. Fourth metasomal tergum melanic. Fifth to eighth metasomal terga melanic. Ovipositor yellowish orange.

♂ unknown

*Etymology*. Bina means ‘wasp’ in Shipibo, an indigenous language of the Peruvian Amazon.

*Holotype*. ♀, PERU, Cusco, Rocotal, 16.ix.2007, Sweep, C. Castillo Leg.

**Figure 3.**
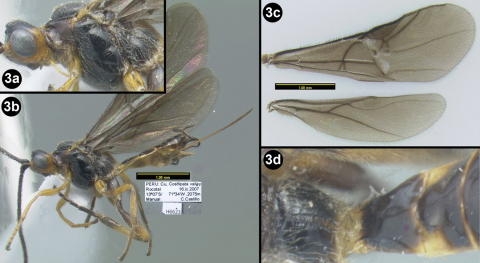
*Sesioctonus bina*. **3a** lateral head and mesosoma **3b** lateral habitus **3c** wings **3d** propodeum and first metasomal segment.

### New Peruvian Distribution Records

***Sesioctonus longinoi***

♀, Cusco, Cosñipata valley, San Pedro, 13°03'23"S, 71°32'55"W, 1520m, Malaise, 7.i.2009,C.Castillo. leg. ♀,Cusco, San Pedro, 13°03'23"S, 71°32'55"W, 1520m, Malaise, C. Castillo. leg.

***Sesioctonus diazi***

♀, Cusco, Reserva Comunal Amarakaeri, Rio Azul, 12°49,8'24"S, 71°05'55"W, 507m, 11.x.2010. C.Castillo. leg. ♀, Loreto, Alto Nanay, Albarenga north, 18M 0533605 9645694, 142m, C. Castillo leg.

## Supplementary Material

XML Treatment for Sesioctonus huggerti

XML Treatment for Sesioctonus wayquecha

XML Treatment for Sesioctonus bina
